# Isotenulin

**DOI:** 10.1107/S1600536813019703

**Published:** 2013-07-20

**Authors:** Kyle S. Knight, Cole T. Smith, Thomas G. Waddell

**Affiliations:** a Department of Chemistry, The University of Tennessee at Chattanooga, Chattanooga, TN 37403, USA

## Abstract

Isotenulin, C_17_H_22_O_5_, is a sesquiterpene lactone isolated from sneezeweed *Helenium amarum*. It crystallizes with two independent mol­ecules in the asymmetric unit. In each mol­ecule, two five-membered rings (cyclo­pentenone and lactone) are *trans*-fused to the central seven-membered ring. The five-membered rings each adopt envelope conformations. The seven-membered ring adopts a twist-chair conformation. In the crystal, the molecules are linked by C—H⋯O interactions, which generate a three-dimensional network.

## Related literature

For the discovery and structural identification of tenulin, see: Clark (1939 ▶); Herz *et al.* (1975 ▶); Braun *et al.* (1956 ▶); Barton *et al.* (1956 ▶). For biological activity that has been observed for tenulin and its analogs, see: Lee *et al.* (1977 ▶); Li *et al.* (2008 ▶); Hodge *et al.* (1995 ▶, and references therein). For the crystal structure of tenulin, see: Knight *et al.* (2013 ▶). For the crystal structure of bromo­isotenulin, see: Mazhar-Ul-Haque *et al.* (1974 ▶).
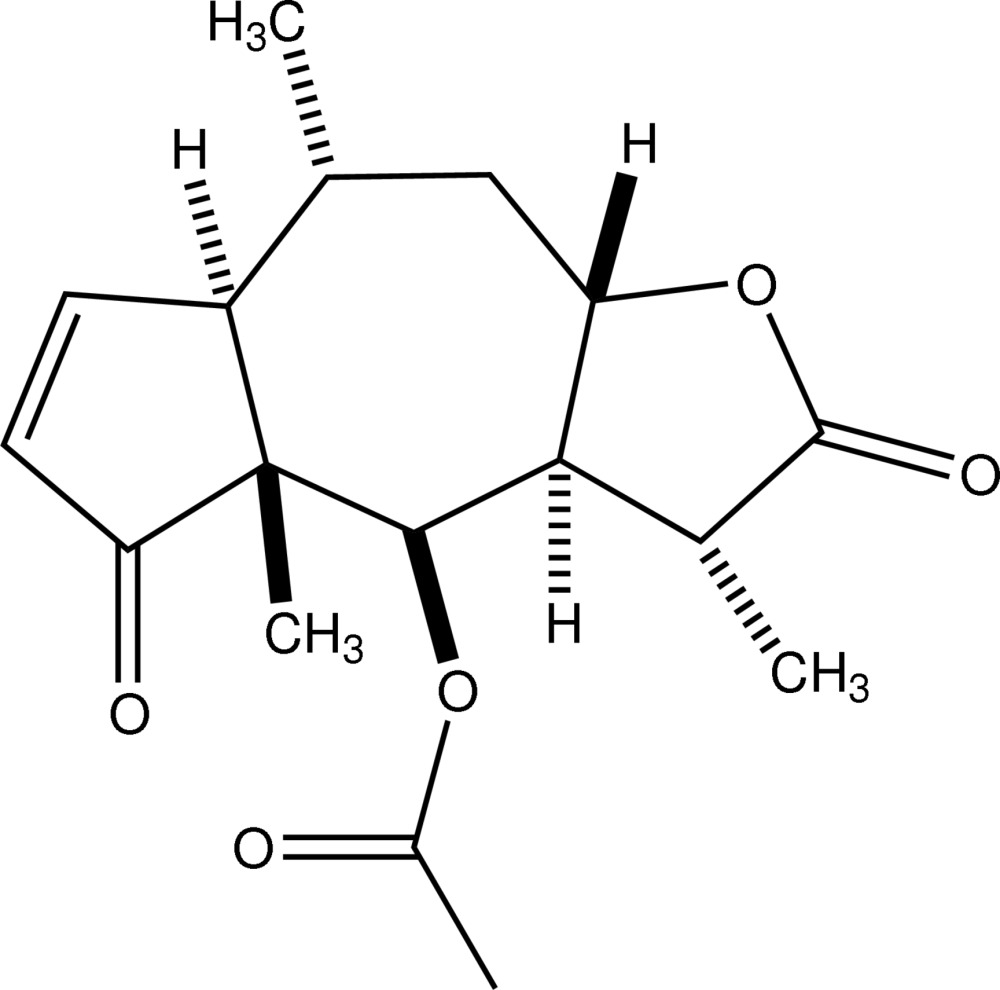


## Experimental

### 

#### Crystal data

C_17_H_22_O_5_
*M* *_r_* = 306.34
Orthorhombic, 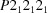

*a* = 6.4565 (11) Å
*b* = 17.625 (3) Å
*c* = 27.997 (4) Å
*V* = 3186.0 (9) Å^3^
*Z* = 8
Mo *K*α radiation
μ = 0.09 mm^−1^
*T* = 200 K
0.55 × 0.3 × 0.2 mm

#### Data collection

Bruker APEXII CCD diffractometer
34729 measured reflections
5611 independent reflections
4883 reflections with *I* > 2σ(*I*)
*R*_int_ = 0.047

#### Refinement

*R*[*F* ^2^ > 2σ(*F* ^2^)] = 0.043
*wR*(*F* ^2^) = 0.100
*S* = 1.01
5611 reflections
405 parameters
H-atom parameters constrained
Δρ_max_ = 0.13 e Å^−3^
Δρ_min_ = −0.17 e Å^−3^

### 

Data collection: *APEX2* (Bruker, 2009 ▶); cell refinement: *SAINT* (Bruker, 2009 ▶); data reduction: *SAINT*; program(s) used to solve structure: *SHELXS97* (Sheldrick, 2008 ▶); program(s) used to refine structure: *SHELXL97* (Sheldrick, 2008 ▶); molecular graphics: *OLEX2* (Dolomanov *et al.*, 2009 ▶); software used to prepare material for publication: *OLEX2*.

## Supplementary Material

Crystal structure: contains datablock(s) global, I. DOI: 10.1107/S1600536813019703/cv5424sup1.cif

Structure factors: contains datablock(s) I. DOI: 10.1107/S1600536813019703/cv5424Isup2.hkl

Supplementary material file. DOI: 10.1107/S1600536813019703/cv5424Isup3.cdx

Additional supplementary materials:  crystallographic information; 3D view; checkCIF report

## Figures and Tables

**Table 1:** Hydrogen-bond geometry (Å, °)

*D*—H⋯*A*	*D*—H	H⋯*A*	*D*⋯*A*	*D*—H⋯*A*
C4—H4⋯O4^i^	1.00	2.50	3.324 (4)	139
C21—H21⋯O5^ii^	1.00	2.44	3.421 (4)	167

Symmetry codes: (i) 
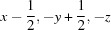
; (ii) 
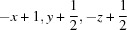
.

**Fig. 1.**
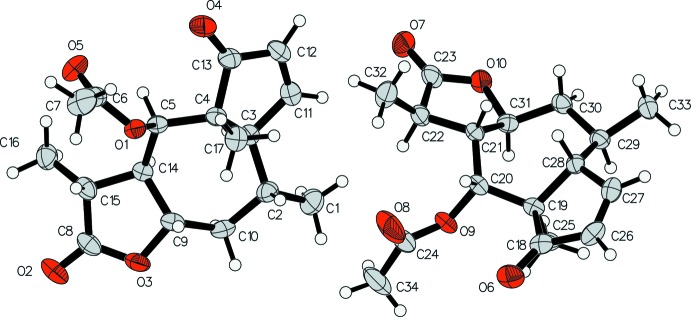
Two independent molecules of isotenulin with the atomic numbering and displacement ellipsoids drawn at the 50% probability level.

## supplementary crystallographic information

### Comment

Isotenulin, C_17_H_22_O_5_, is a sesquiterpene lactone isolated from sneezeweed *Helenium amarum*, a medicinal plant native to the southeastern USA. The crystal structure contains two independent tenulin molecules. These two conformations are distinguished by the way in which the five membered rings twist relative to each other, as measured by the magnitude of the dihedral angles at the cycloheptane-cyclopentenone and cycloheptane-lactone ring junctures. In the more twisted conformation, the dihedral angle of the substituents on the cycloheptane ring that form the cyclopentenone is 21.5 (1)°, while that of the lactone is 30.2 (1)°. Those values for the less twisted conformer are 18.1 (1)° and 28.2 (1)° respectively. The absolute configuration of all other stereocenters is that established for other sesquiterpene lactones (Mazhar-Ul-Haque *et al.*, 1974).

### Experimental

Isotenulin was prepared as described previously (Hodge *et al.*, 1995).

### Refinement

H atoms were positioned geometrically at bond distances of 0.98, 0.99, 1.00 and 0.95 Å for methyl, methylene, methine and vinyl, respectively, and constrained to ride on their parent atoms with *U*_iso_(H)= 1.2–1.5 *U*_eq_(C).

### Crystal data

**Table:**

C_17_H_22_O_5_	*D*_x_ = 1.277 Mg m^−^^3^
*M**_r_* = 306.34	Mo *K*α radiation, λ = 0.71073 Å
Orthorhombic, *P*2_1_2_1_2_1_	Cell parameters from 9402 reflections
*a* = 6.4565 (11) Å	θ = 2.3–23.8°
*b* = 17.625 (3) Å	µ = 0.09 mm^−^^1^
*c* = 27.997 (4) Å	*T* = 200 K
*V* = 3186.0 (9) Å^3^	Block, colourless
*Z* = 8	0.55 × 0.3 × 0.2 mm
*F*(000) = 1312	

### Data collection

**Table:**

Bruker APEXII CCD diffractometer	*R*_int_ = 0.047
Graphite monochromator	θ_max_ = 25.1°, θ_min_ = 2.3°
φ and ω scans	*h* = −7→7
34729 measured reflections	*k* = −21→20
5611 independent reflections	*l* = −31→31
4883 reflections with *I* > 2σ(*I*)	

### Refinement

**Table:**

Refinement on *F*^2^	0 restraints
Least-squares matrix: full	Hydrogen site location: inferred from neighbouring sites
*R*[*F*^2^ > 2σ(*F*^2^)] = 0.043	H-atom parameters constrained
*wR*(*F*^2^) = 0.100	*w* = 1/[σ^2^(*F*_o_^2^) + (0.0573*P*)^2^ + 0.5452*P*] where *P* = (*F*_o_^2^ + 2*F*_c_^2^)/3
*S* = 1.01	(Δ/σ)_max_ < 0.001
5611 reflections	Δρ_max_ = 0.13 e Å^−^^3^
405 parameters	Δρ_min_ = −0.17 e Å^−^^3^

### Special details

**Table:**

Geometry. All e.s.d.'s (except the e.s.d. in the dihedral angle between two l.s. planes) are estimated using the full covariance matrix. The cell e.s.d.'s are taken into account individually in the estimation of e.s.d.'s in distances, angles and torsion angles; correlations between e.s.d.'s in cell parameters are only used when they are defined by crystal symmetry. An approximate (isotropic) treatment of cell e.s.d.'s is used for estimating e.s.d.'s involving l.s. planes.

### Fractional atomic coordinates and isotropic or equivalent isotropic displacement parameters (Å^2^)

**Table:**

	*x*	*y*	*z*	*U*_iso_*/*U*_eq_	
O1	0.6652 (3)	0.32092 (12)	0.13792 (7)	0.0282 (5)	
O4	0.4353 (4)	0.20905 (15)	−0.02363 (8)	0.0460 (6)	
O2	0.4385 (4)	0.33063 (13)	−0.00295 (7)	0.0384 (6)	
O5	0.4353 (5)	0.39087 (15)	0.23006 (8)	0.0521 (7)	
O3	0.5852 (4)	0.21748 (14)	0.18164 (8)	0.0437 (6)	
O10	0.8514 (4)	0.91154 (14)	0.06402 (9)	0.0426 (6)	
O9	0.0773 (4)	0.66834 (13)	0.21828 (9)	0.0470 (6)	
O8	0.8155 (5)	0.72399 (18)	0.09136 (11)	0.0705 (9)	
O6	0.5119 (3)	0.78613 (12)	0.08363 (7)	0.0322 (5)	
O7	0.0566 (3)	0.78753 (12)	0.19284 (8)	0.0342 (5)	
C17	0.1123 (6)	0.5589 (2)	0.05783 (14)	0.0489 (10)	
H17A	0.1440	0.5799	0.0263	0.073*	
H17B	0.1065	0.6000	0.0813	0.073*	
H17C	−0.0218	0.5329	0.0567	0.073*	
C14	0.2811 (5)	0.50237 (18)	0.07211 (11)	0.0333 (8)	
H14	0.4150	0.5305	0.0750	0.040*	
C13	0.2347 (5)	0.46402 (17)	0.12044 (10)	0.0267 (7)	
H13	0.1279	0.4241	0.1142	0.032*	
C9	0.4208 (5)	0.42363 (16)	0.14536 (10)	0.0256 (7)	
C3	0.4490 (5)	0.33964 (16)	0.13131 (10)	0.0241 (6)	
H3	0.3655	0.3079	0.1538	0.029*	
C2	0.7118 (5)	0.26042 (19)	0.16595 (12)	0.0331 (7)	
C8	0.9396 (6)	0.2557 (2)	0.17373 (14)	0.0541 (10)	
H8A	0.9851	0.2990	0.1930	0.081*	
H8B	1.0108	0.2566	0.1428	0.081*	
H8C	0.9728	0.2084	0.1905	0.081*	
C6	0.4421 (5)	0.2565 (2)	0.00761 (12)	0.0355 (8)	
C5	0.4467 (5)	0.37680 (18)	0.04092 (10)	0.0316 (7)	
H5	0.5910	0.3951	0.0468	0.038*	
C15	0.3031 (5)	0.44332 (19)	0.03186 (11)	0.0355 (8)	
H15A	0.1636	0.4229	0.0247	0.043*	
H15B	0.3520	0.4700	0.0028	0.043*	
C12	0.1511 (5)	0.51352 (18)	0.16020 (12)	0.0329 (7)	
H12	0.0591	0.5547	0.1549	0.040*	
C11	0.2185 (5)	0.49380 (19)	0.20298 (12)	0.0363 (8)	
H11	0.1781	0.5170	0.2322	0.044*	
C10	0.3644 (5)	0.43101 (18)	0.19861 (11)	0.0327 (7)	
C4	0.3809 (4)	0.32060 (16)	0.08008 (10)	0.0253 (6)	
H4	0.2260	0.3190	0.0800	0.030*	
C1	0.4563 (5)	0.24438 (18)	0.06090 (11)	0.0324 (7)	
H1	0.6058	0.2388	0.0695	0.039*	
C7	0.3428 (6)	0.17418 (18)	0.07783 (12)	0.0435 (9)	
H7A	0.1950	0.1791	0.0706	0.065*	
H7B	0.3613	0.1685	0.1124	0.065*	
H7C	0.3987	0.1295	0.0615	0.065*	
C16	0.6230 (5)	0.46954 (18)	0.14028 (13)	0.0364 (8)	
H16A	0.5950	0.5232	0.1468	0.055*	
H16B	0.6765	0.4641	0.1077	0.055*	
H16C	0.7258	0.4505	0.1631	0.055*	
C27	0.7412 (5)	0.94561 (18)	0.09205 (11)	0.0300 (7)	
C26	0.5369 (4)	0.91551 (16)	0.11260 (10)	0.0241 (6)	
C20	0.5510 (5)	0.83112 (16)	0.12654 (9)	0.0247 (6)	
H20	0.6953	0.8205	0.1377	0.030*	
C21	0.4003 (4)	0.80785 (16)	0.16622 (10)	0.0245 (6)	
H21	0.4636	0.8252	0.1969	0.029*	
C18	0.3614 (5)	0.72245 (17)	0.17173 (11)	0.0306 (7)	
H18	0.3445	0.6999	0.1392	0.037*	
C23	0.1542 (5)	0.72034 (18)	0.19676 (12)	0.0344 (8)	
C19	0.6589 (6)	0.7361 (2)	0.06999 (13)	0.0448 (9)	
C33	0.3809 (5)	0.93110 (19)	0.07173 (10)	0.0317 (7)	
H33A	0.3752	0.9857	0.0653	0.048*	
H33B	0.2432	0.9131	0.0812	0.048*	
H33C	0.4255	0.9043	0.0428	0.048*	
C28	0.7730 (5)	1.02207 (18)	0.11142 (12)	0.0351 (8)	
H28	0.8739	1.0573	0.1005	0.042*	
C29	0.6391 (5)	1.03451 (17)	0.14657 (12)	0.0329 (7)	
H29	0.6320	1.0810	0.1636	0.039*	
C30	0.4996 (4)	0.96816 (16)	0.15690 (10)	0.0256 (7)	
H30	0.5592	0.9414	0.1852	0.031*	
C31	0.2724 (5)	0.98620 (17)	0.16899 (11)	0.0290 (7)	
H31	0.2009	1.0018	0.1389	0.035*	
C32	0.1562 (5)	0.91723 (17)	0.18982 (11)	0.0294 (7)	
H32A	0.2012	0.9109	0.2234	0.035*	
H32B	0.0067	0.9297	0.1904	0.035*	
C22	0.1807 (5)	0.84120 (17)	0.16491 (11)	0.0277 (7)	
H22	0.1287	0.8441	0.1313	0.033*	
C24	0.5304 (6)	0.67908 (19)	0.19779 (13)	0.0439 (9)	
H24A	0.5580	0.7034	0.2286	0.066*	
H24B	0.6571	0.6792	0.1785	0.066*	
H24C	0.4853	0.6267	0.2031	0.066*	
C34	0.2531 (6)	1.05118 (18)	0.20498 (13)	0.0431 (9)	
H34A	0.3292	1.0382	0.2341	0.065*	
H34B	0.1067	1.0593	0.2127	0.065*	
H34C	0.3110	1.0976	0.1911	0.065*	
C25	0.5960 (8)	0.6984 (2)	0.02392 (14)	0.0628 (12)	
H25A	0.6992	0.6600	0.0153	0.094*	
H25B	0.5874	0.7365	−0.0015	0.094*	
H25C	0.4606	0.6741	0.0280	0.094*	

### Atomic displacement parameters (Å^2^)

**Table:**

	*U*^11^	*U*^22^	*U*^33^	*U*^12^	*U*^13^	*U*^23^
O1	0.0238 (10)	0.0339 (11)	0.0271 (11)	0.0024 (9)	0.0006 (9)	0.0045 (10)
O4	0.0406 (14)	0.0598 (16)	0.0378 (13)	0.0078 (13)	0.0003 (12)	−0.0218 (12)
O2	0.0446 (14)	0.0484 (15)	0.0224 (11)	0.0033 (12)	0.0045 (10)	−0.0046 (10)
O5	0.0764 (19)	0.0547 (15)	0.0253 (12)	0.0087 (15)	−0.0102 (13)	−0.0023 (12)
O3	0.0402 (14)	0.0408 (13)	0.0500 (15)	−0.0044 (12)	−0.0046 (12)	0.0171 (11)
O10	0.0275 (12)	0.0566 (16)	0.0438 (14)	0.0042 (11)	0.0089 (11)	0.0024 (12)
O9	0.0493 (15)	0.0327 (13)	0.0590 (15)	−0.0097 (12)	0.0163 (13)	0.0010 (12)
O8	0.063 (2)	0.086 (2)	0.0628 (19)	0.0438 (17)	−0.0031 (16)	−0.0259 (17)
O6	0.0304 (12)	0.0382 (12)	0.0280 (11)	0.0024 (9)	0.0029 (9)	−0.0120 (10)
O7	0.0278 (11)	0.0319 (12)	0.0429 (13)	−0.0028 (10)	0.0088 (10)	−0.0016 (10)
C17	0.059 (2)	0.044 (2)	0.044 (2)	0.0129 (19)	−0.0084 (19)	0.0053 (17)
C14	0.0392 (18)	0.0320 (16)	0.0285 (18)	−0.0013 (14)	−0.0037 (14)	0.0026 (14)
C13	0.0236 (15)	0.0277 (16)	0.0288 (17)	−0.0016 (13)	−0.0004 (13)	−0.0022 (13)
C9	0.0238 (15)	0.0302 (15)	0.0228 (15)	−0.0024 (13)	−0.0026 (13)	−0.0015 (13)
C3	0.0186 (14)	0.0303 (16)	0.0234 (15)	−0.0026 (12)	0.0013 (12)	0.0022 (12)
C2	0.0357 (18)	0.0369 (18)	0.0267 (16)	0.0038 (15)	0.0011 (15)	0.0015 (15)
C8	0.035 (2)	0.070 (3)	0.057 (2)	0.016 (2)	−0.0001 (18)	0.019 (2)
C6	0.0241 (16)	0.048 (2)	0.0341 (18)	0.0061 (16)	0.0003 (14)	−0.0122 (17)
C5	0.0324 (16)	0.0404 (18)	0.0220 (15)	−0.0027 (15)	0.0017 (14)	−0.0022 (14)
C15	0.042 (2)	0.0411 (19)	0.0234 (17)	−0.0022 (16)	−0.0004 (14)	0.0082 (14)
C12	0.0257 (15)	0.0316 (16)	0.042 (2)	−0.0012 (14)	0.0022 (15)	−0.0049 (15)
C11	0.0374 (19)	0.0398 (19)	0.0318 (19)	−0.0045 (16)	0.0051 (15)	−0.0116 (15)
C10	0.0355 (18)	0.0369 (18)	0.0256 (17)	−0.0076 (15)	−0.0063 (15)	−0.0037 (15)
C4	0.0198 (14)	0.0303 (16)	0.0259 (15)	−0.0009 (12)	0.0001 (12)	−0.0010 (13)
C1	0.0292 (16)	0.0366 (17)	0.0314 (16)	0.0091 (15)	−0.0046 (14)	−0.0071 (14)
C7	0.058 (2)	0.0315 (18)	0.041 (2)	0.0013 (17)	−0.0110 (18)	−0.0045 (16)
C16	0.0296 (17)	0.0351 (17)	0.044 (2)	−0.0045 (15)	−0.0028 (16)	−0.0010 (16)
C27	0.0206 (15)	0.0419 (18)	0.0275 (17)	0.0050 (14)	−0.0042 (14)	0.0056 (15)
C26	0.0170 (14)	0.0342 (17)	0.0210 (15)	0.0031 (13)	−0.0050 (12)	−0.0019 (13)
C20	0.0189 (14)	0.0304 (16)	0.0247 (15)	0.0025 (13)	−0.0031 (12)	−0.0071 (13)
C21	0.0256 (15)	0.0258 (14)	0.0221 (15)	−0.0002 (12)	−0.0017 (13)	−0.0049 (12)
C18	0.0335 (17)	0.0277 (15)	0.0306 (17)	−0.0016 (14)	0.0029 (14)	−0.0059 (14)
C23	0.0380 (18)	0.0299 (17)	0.0353 (18)	−0.0071 (16)	0.0027 (16)	−0.0099 (15)
C19	0.050 (2)	0.043 (2)	0.041 (2)	0.0078 (18)	0.0134 (19)	−0.0092 (17)
C33	0.0275 (16)	0.0452 (19)	0.0224 (17)	0.0022 (14)	−0.0025 (13)	−0.0002 (14)
C28	0.0246 (16)	0.0370 (18)	0.0436 (19)	−0.0042 (14)	−0.0036 (15)	0.0138 (16)
C29	0.0300 (16)	0.0282 (16)	0.0405 (19)	0.0006 (14)	−0.0107 (15)	0.0021 (14)
C30	0.0282 (16)	0.0245 (15)	0.0241 (16)	0.0030 (12)	−0.0053 (12)	0.0001 (12)
C31	0.0299 (16)	0.0312 (16)	0.0259 (16)	0.0050 (13)	0.0015 (14)	0.0007 (14)
C32	0.0253 (15)	0.0330 (16)	0.0299 (17)	0.0064 (13)	0.0041 (14)	−0.0017 (13)
C22	0.0256 (16)	0.0320 (16)	0.0256 (15)	−0.0030 (13)	0.0027 (13)	0.0008 (13)
C24	0.041 (2)	0.0335 (18)	0.057 (2)	0.0031 (17)	0.0045 (18)	0.0076 (18)
C34	0.057 (2)	0.0284 (17)	0.044 (2)	0.0022 (17)	0.0137 (18)	−0.0048 (16)
C25	0.076 (3)	0.062 (3)	0.051 (2)	0.003 (2)	0.015 (2)	−0.030 (2)

### Geometric parameters (Å, º)

**Table:**

O1—C3	1.446 (3)	C1—C7	1.514 (5)
O1—C2	1.358 (4)	C7—H7A	0.9800
O4—C6	1.211 (4)	C7—H7B	0.9800
O2—C6	1.340 (4)	C7—H7C	0.9800
O2—C5	1.474 (4)	C16—H16A	0.9800
O5—C10	1.219 (4)	C16—H16B	0.9800
O3—C2	1.198 (4)	C16—H16C	0.9800
O10—C27	1.218 (4)	C27—C26	1.533 (4)
O9—C23	1.204 (4)	C27—C28	1.467 (5)
O8—C19	1.194 (5)	C26—C20	1.540 (4)
O6—C20	1.462 (3)	C26—C33	1.549 (4)
O6—C19	1.350 (4)	C26—C30	1.568 (4)
O7—C23	1.346 (4)	C20—H20	1.0000
O7—C22	1.466 (4)	C20—C21	1.533 (4)
C17—H17A	0.9800	C21—H21	1.0000
C17—H17B	0.9800	C21—C18	1.534 (4)
C17—H17C	0.9800	C21—C22	1.535 (4)
C17—C14	1.530 (5)	C18—H18	1.0000
C14—H14	1.0000	C18—C23	1.511 (5)
C14—C13	1.542 (4)	C18—C24	1.519 (5)
C14—C15	1.540 (5)	C19—C25	1.507 (5)
C13—H13	1.0000	C33—H33A	0.9800
C13—C9	1.561 (4)	C33—H33B	0.9800
C13—C12	1.514 (4)	C33—H33C	0.9800
C9—C3	1.542 (4)	C28—H28	0.9500
C9—C10	1.540 (4)	C28—C29	1.328 (5)
C9—C16	1.542 (4)	C29—H29	0.9500
C3—H3	1.0000	C29—C30	1.504 (4)
C3—C4	1.537 (4)	C30—H30	1.0000
C2—C8	1.489 (5)	C30—C31	1.539 (4)
C8—H8A	0.9800	C31—H31	1.0000
C8—H8B	0.9800	C31—C32	1.543 (4)
C8—H8C	0.9800	C31—C34	1.530 (4)
C6—C1	1.510 (5)	C32—H32A	0.9900
C5—H5	1.0000	C32—H32B	0.9900
C5—C15	1.516 (5)	C32—C22	1.519 (4)
C5—C4	1.537 (4)	C22—H22	1.0000
C15—H15A	0.9900	C24—H24A	0.9800
C15—H15B	0.9900	C24—H24B	0.9800
C12—H12	0.9500	C24—H24C	0.9800
C12—C11	1.321 (5)	C34—H34A	0.9800
C11—H11	0.9500	C34—H34B	0.9800
C11—C10	1.459 (5)	C34—H34C	0.9800
C4—H4	1.0000	C25—H25A	0.9800
C4—C1	1.526 (4)	C25—H25B	0.9800
C1—H1	1.0000	C25—H25C	0.9800
			
C2—O1—C3	117.8 (2)	H16A—C16—H16C	109.5
C6—O2—C5	110.7 (2)	H16B—C16—H16C	109.5
C19—O6—C20	117.7 (3)	O10—C27—C26	125.0 (3)
C23—O7—C22	110.8 (2)	O10—C27—C28	127.6 (3)
H17A—C17—H17B	109.5	C28—C27—C26	107.4 (3)
H17A—C17—H17C	109.5	C27—C26—C20	112.2 (2)
H17B—C17—H17C	109.5	C27—C26—C33	102.8 (2)
C14—C17—H17A	109.5	C27—C26—C30	103.0 (2)
C14—C17—H17B	109.5	C20—C26—C33	113.4 (2)
C14—C17—H17C	109.5	C20—C26—C30	112.3 (2)
C17—C14—H14	108.3	C33—C26—C30	112.3 (2)
C17—C14—C13	112.1 (3)	O6—C20—C26	107.8 (2)
C17—C14—C15	108.3 (3)	O6—C20—H20	108.4
C13—C14—H14	108.3	O6—C20—C21	109.9 (2)
C15—C14—H14	108.3	C26—C20—H20	108.4
C15—C14—C13	111.3 (3)	C21—C20—C26	113.9 (2)
C14—C13—H13	106.8	C21—C20—H20	108.4
C14—C13—C9	116.3 (3)	C20—C21—H21	106.3
C9—C13—H13	106.8	C20—C21—C18	116.1 (2)
C12—C13—C14	117.5 (3)	C20—C21—C22	117.8 (2)
C12—C13—H13	106.8	C18—C21—H21	106.3
C12—C13—C9	102.0 (2)	C18—C21—C22	103.1 (2)
C3—C9—C13	114.5 (2)	C22—C21—H21	106.3
C10—C9—C13	102.3 (2)	C21—C18—H18	108.4
C10—C9—C3	110.8 (2)	C23—C18—C21	102.5 (2)
C10—C9—C16	104.2 (2)	C23—C18—H18	108.4
C16—C9—C13	111.8 (2)	C23—C18—C24	113.6 (3)
C16—C9—C3	112.3 (2)	C24—C18—C21	115.1 (3)
O1—C3—C9	107.5 (2)	C24—C18—H18	108.4
O1—C3—H3	108.2	O9—C23—O7	121.2 (3)
O1—C3—C4	110.2 (2)	O9—C23—C18	128.0 (3)
C9—C3—H3	108.2	O7—C23—C18	110.8 (3)
C4—C3—C9	114.4 (2)	O8—C19—O6	124.8 (3)
C4—C3—H3	108.2	O8—C19—C25	125.3 (3)
O1—C2—C8	110.3 (3)	O6—C19—C25	109.9 (4)
O3—C2—O1	123.8 (3)	C26—C33—H33A	109.5
O3—C2—C8	125.8 (3)	C26—C33—H33B	109.5
C2—C8—H8A	109.5	C26—C33—H33C	109.5
C2—C8—H8B	109.5	H33A—C33—H33B	109.5
C2—C8—H8C	109.5	H33A—C33—H33C	109.5
H8A—C8—H8B	109.5	H33B—C33—H33C	109.5
H8A—C8—H8C	109.5	C27—C28—H28	125.2
H8B—C8—H8C	109.5	C29—C28—C27	109.5 (3)
O4—C6—O2	120.9 (3)	C29—C28—H28	125.2
O4—C6—C1	128.2 (3)	C28—C29—H29	123.1
O2—C6—C1	110.9 (3)	C28—C29—C30	113.8 (3)
O2—C5—H5	110.4	C30—C29—H29	123.1
O2—C5—C15	105.4 (2)	C26—C30—H30	106.8
O2—C5—C4	103.2 (2)	C29—C30—C26	102.5 (2)
C15—C5—H5	110.4	C29—C30—H30	106.8
C15—C5—C4	116.7 (3)	C29—C30—C31	116.9 (2)
C4—C5—H5	110.4	C31—C30—C26	116.3 (2)
C14—C15—H15A	108.0	C31—C30—H30	106.8
C14—C15—H15B	108.0	C30—C31—H31	108.1
C5—C15—C14	117.2 (3)	C30—C31—C32	112.6 (2)
C5—C15—H15A	108.0	C32—C31—H31	108.1
C5—C15—H15B	108.0	C34—C31—C30	112.1 (3)
H15A—C15—H15B	107.2	C34—C31—H31	108.1
C13—C12—H12	123.3	C34—C31—C32	107.5 (3)
C11—C12—C13	113.4 (3)	C31—C32—H32A	107.8
C11—C12—H12	123.3	C31—C32—H32B	107.8
C12—C11—H11	125.2	H32A—C32—H32B	107.1
C12—C11—C10	109.7 (3)	C22—C32—C31	118.1 (2)
C10—C11—H11	125.2	C22—C32—H32A	107.8
O5—C10—C9	124.2 (3)	C22—C32—H32B	107.8
O5—C10—C11	128.5 (3)	O7—C22—C21	104.2 (2)
C11—C10—C9	107.3 (3)	O7—C22—C32	105.5 (2)
C3—C4—C5	116.5 (2)	O7—C22—H22	110.6
C3—C4—H4	107.1	C21—C22—H22	110.6
C5—C4—H4	107.1	C32—C22—C21	115.0 (3)
C1—C4—C3	115.4 (2)	C32—C22—H22	110.6
C1—C4—C5	103.2 (2)	C18—C24—H24A	109.5
C1—C4—H4	107.1	C18—C24—H24B	109.5
C6—C1—C4	101.8 (2)	C18—C24—H24C	109.5
C6—C1—H1	108.1	H24A—C24—H24B	109.5
C6—C1—C7	113.3 (3)	H24A—C24—H24C	109.5
C4—C1—H1	108.1	H24B—C24—H24C	109.5
C7—C1—C4	117.0 (3)	C31—C34—H34A	109.5
C7—C1—H1	108.1	C31—C34—H34B	109.5
C1—C7—H7A	109.5	C31—C34—H34C	109.5
C1—C7—H7B	109.5	H34A—C34—H34B	109.5
C1—C7—H7C	109.5	H34A—C34—H34C	109.5
H7A—C7—H7B	109.5	H34B—C34—H34C	109.5
H7A—C7—H7C	109.5	C19—C25—H25A	109.5
H7B—C7—H7C	109.5	C19—C25—H25B	109.5
C9—C16—H16A	109.5	C19—C25—H25C	109.5
C9—C16—H16B	109.5	H25A—C25—H25B	109.5
C9—C16—H16C	109.5	H25A—C25—H25C	109.5
H16A—C16—H16B	109.5	H25B—C25—H25C	109.5
			
O1—C3—C4—C5	−77.5 (3)	C10—C9—C3—C4	146.2 (3)
O1—C3—C4—C1	43.8 (3)	C4—C5—C15—C14	66.5 (4)
O4—C6—C1—C4	−160.3 (3)	C16—C9—C3—O1	25.0 (3)
O4—C6—C1—C7	−33.7 (5)	C16—C9—C3—C4	−97.7 (3)
O2—C6—C1—C4	20.1 (3)	C16—C9—C10—O5	−84.0 (4)
O2—C6—C1—C7	146.6 (3)	C16—C9—C10—C11	95.2 (3)
O2—C5—C15—C14	−179.7 (3)	C27—C26—C20—O6	−84.3 (3)
O2—C5—C4—C3	157.7 (2)	C27—C26—C20—C21	153.5 (2)
O2—C5—C4—C1	30.2 (3)	C27—C26—C30—C29	18.1 (3)
O10—C27—C26—C20	41.9 (4)	C27—C26—C30—C31	146.9 (3)
O10—C27—C26—C33	−80.3 (3)	C27—C28—C29—C30	1.1 (4)
O10—C27—C26—C30	162.9 (3)	C26—C27—C28—C29	11.6 (3)
O10—C27—C28—C29	−169.9 (3)	C26—C20—C21—C18	163.0 (2)
O6—C20—C21—C18	42.0 (3)	C26—C20—C21—C22	40.0 (3)
O6—C20—C21—C22	−81.0 (3)	C26—C30—C31—C32	71.5 (3)
C17—C14—C13—C9	−163.2 (3)	C26—C30—C31—C34	−167.1 (3)
C17—C14—C13—C12	−42.0 (4)	C20—O6—C19—O8	2.8 (5)
C17—C14—C15—C5	−173.7 (3)	C20—O6—C19—C25	−177.0 (3)
C14—C13—C9—C3	−89.3 (3)	C20—C26—C30—C29	139.1 (2)
C14—C13—C9—C10	150.7 (3)	C20—C26—C30—C31	−92.2 (3)
C14—C13—C9—C16	39.8 (4)	C20—C21—C18—C23	−157.5 (2)
C14—C13—C12—C11	−144.6 (3)	C20—C21—C18—C24	78.6 (3)
C13—C14—C15—C5	−50.0 (4)	C20—C21—C22—O7	157.5 (2)
C13—C9—C3—O1	153.9 (2)	C20—C21—C22—C32	−87.5 (3)
C13—C9—C3—C4	31.1 (3)	C21—C18—C23—O9	−162.1 (3)
C13—C9—C10—O5	159.5 (3)	C21—C18—C23—O7	17.2 (3)
C13—C9—C10—C11	−21.3 (3)	C18—C21—C22—O7	28.2 (3)
C13—C12—C11—C10	2.7 (4)	C18—C21—C22—C32	143.2 (3)
C9—C13—C12—C11	−16.2 (3)	C23—O7—C22—C21	−18.7 (3)
C9—C3—C4—C5	43.8 (3)	C23—O7—C22—C32	−140.3 (3)
C9—C3—C4—C1	165.0 (3)	C19—O6—C20—C26	122.9 (3)
C3—O1—C2—O3	7.6 (4)	C19—O6—C20—C21	−112.5 (3)
C3—O1—C2—C8	−172.7 (3)	C33—C26—C20—O6	31.6 (3)
C3—C9—C10—O5	37.0 (4)	C33—C26—C20—C21	−90.6 (3)
C3—C9—C10—C11	−143.8 (3)	C33—C26—C30—C29	−91.7 (3)
C3—C4—C1—C6	−158.2 (3)	C33—C26—C30—C31	37.0 (4)
C3—C4—C1—C7	77.8 (4)	C28—C27—C26—C20	−139.5 (2)
C2—O1—C3—C9	125.7 (3)	C28—C27—C26—C33	98.3 (3)
C2—O1—C3—C4	−109.0 (3)	C28—C27—C26—C30	−18.5 (3)
C6—O2—C5—C15	−141.7 (3)	C28—C29—C30—C26	−12.8 (3)
C6—O2—C5—C4	−18.8 (3)	C28—C29—C30—C31	−141.1 (3)
C5—O2—C6—O4	179.6 (3)	C29—C30—C31—C32	−167.1 (3)
C5—O2—C6—C1	−0.8 (4)	C29—C30—C31—C34	−45.7 (4)
C5—C4—C1—C6	−30.0 (3)	C30—C26—C20—O6	160.3 (2)
C5—C4—C1—C7	−154.1 (3)	C30—C26—C20—C21	38.0 (3)
C15—C14—C13—C9	75.2 (3)	C30—C31—C32—C22	−46.3 (4)
C15—C14—C13—C12	−163.5 (3)	C31—C32—C22—O7	179.2 (3)
C15—C5—C4—C3	−87.3 (3)	C31—C32—C22—C21	65.0 (4)
C15—C5—C4—C1	145.2 (3)	C22—O7—C23—O9	−179.7 (3)
C12—C13—C9—C3	141.5 (3)	C22—O7—C23—C18	0.9 (3)
C12—C13—C9—C10	21.5 (3)	C22—C21—C18—C23	−27.1 (3)
C12—C13—C9—C16	−89.4 (3)	C22—C21—C18—C24	−151.0 (3)
C12—C11—C10—O5	−168.5 (4)	C24—C18—C23—O9	−37.3 (5)
C12—C11—C10—C9	12.4 (4)	C24—C18—C23—O7	142.1 (3)
C10—C9—C3—O1	−91.1 (3)	C34—C31—C32—C22	−170.3 (3)

### Hydrogen-bond geometry (Å, º)

**Table:**

*D*—H···*A*	*D*—H	H···*A*	*D*···*A*	*D*—H···*A*
C4—H4···O4^i^	1.00	2.50	3.324 (4)	139
C21—H21···O5^ii^	1.00	2.44	3.421 (4)	167

Symmetry codes: (i) *x*−1/2, −*y*+1/2, −*z*; (ii) −*x*+1, *y*+1/2, −*z*+1/2.

## References

[bb1] Barton, D. H. R. & De Mayo, P. (1956). *J. Chem. Soc.* pp. 142–149.

[bb2] Braun, B. H., Herz, W. & Rabindran, K. (1956). *J. Am. Chem. Soc.* **78**, 4423–4429.

[bb3] Bruker (2009). *APEX2* and *SAINT* Bruker AXS Inc., Madison, Wisconsin, USA.

[bb4] Clark, E. P. (1939). *J. Am. Chem. Soc.* **61**, 1836–1840.

[bb5] Dolomanov, O. V., Bourhis, L. J., Gildea, R. J., Howard, J. A. K. & Puschmann, H. (2009). *J. Appl. Cryst.* **42**, 339–341.

[bb6] Herz, W. & Sharma, R. P. (1975). *J. Org. Chem.* **40**, 2557–2559.

[bb7] Hodge, J. S. & Waddell, T. G. (1995). *J. Nat. Prod.* **58**, 149–151.

[bb8] Knight, K. S., Smith, C. T., Waddell, T. G. & Noll, B. (2013). *Acta Cryst.* E**69**, o1237–o1238. 10.1107/S1600536813018369 PMC3793741 24109328

[bb9] Lee, K. H., Hall, I. H., Mar, E. C., Starnes, C. O., ElGebaly, S. A., Waddell, T. G., Hadgraft, R. I., Ruffner, C. G. & Weidner, I. (1977). *Science*, **196**, 533–536. 10.1126/science.191909 191909

[bb10] Li, X.-J. & Zhang, H.-Y. (2008). *Trends Mol. Med.* **14**, 1–2. 10.1016/j.molmed.2007.11.002 18054521

[bb11] Mazhar-Ul-Haque, Rogers, D. & Caughlan, C. N. (1974). *J. Chem. Soc. Perkin Trans. 2*, pp. 223–228.

[bb12] Sheldrick, G. M. (2008). *Acta Cryst.* A**64**, 112–122. 10.1107/S0108767307043930 18156677

